# 三代后的新一代EGFR-TKIs研究进展

**DOI:** 10.3779/j.issn.1009-3419.2020.102.28

**Published:** 2020-11-20

**Authors:** 媛媛 刘, 义慧 李, 建功 王

**Affiliations:** 063000 唐山，华北理工大学附属唐山市人民医院肿瘤综合治疗一科 First Department of Comprehensive Treatment of Tumors, Tangshan People's Hospital, North China University of Science and Technology, Tangshan 063000, China

**Keywords:** 肺肿瘤, 新一代, 表皮生长因子受体酪氨酸激酶抑制剂, Lung neoplasms, New generation, Epidermal growth factor receptor-tyrosine kinase inhibitors

## Abstract

肺癌是全球死亡率最高的癌种。第一、二代表皮生长因子受体酪氨酸激酶抑制剂（epidermal growth factor receptor-tyrosine kinase inhibitors, EGFR-TKIs）的出现，在一定程度上极大地提高了非小细胞肺癌（non-small cell lung cancer, NSCLC）患者的生存期及生活质量，但大多数患者在经过一段时间的无进展生存期后会产生耐药性，其中以T790M突变为主要耐药机制。针对此耐药突变出现的是以奥希替尼为代表的第三代EGFR-TKIs，其效果显著，然而仍不可避免的出现耐药性，如：C797S突变、间质表皮转化（mesenchymal-epithelial transition, MET）、*RAS*突变、*BRAF*突变、小细胞肺癌（small cell lung cancer, SCLC）转化、上皮间质细胞转化（epithelial mesenchymal transition, EMT）等。但是目前第三代EGFR-TKIs耐药后并没有标准有效的治疗方案。故本文主要阐述三代后的新一代EGFR-TKIs的研究进展，为后续的研究及治疗提供一定的参考。

## Abstract (en)

Lung cancer has the highest mortality rate in the world. The first- and second-generation epidermal growth factor receptor-tyrosine kinase inhibitors (EGFR-TKIs) greatly improve the survival time and quality of life of patients with non-small cell lung cancer (NSCLC) to some extent. However, after a period of progression-free survival, most patients develop drug resistance, in which T790M mutation is the mainly resistance mechanism. The third-generation EGFR-TKIs, represented by osimertinib, are found to have significant effect on this resistance. The effect is remarkable, but drug resistance is still inevitable. For example, C797S mutation, mesenchymal-epithelial transition (MET), *RAS* mutation, *BRAF* mutation, transformation of small cell lung cancer (SCLC), transformation of epithelial mesenchymal transition (EMT), *etc*. But, there is no standard and effective treatment after the third-generation EGFR-TKIs resistance. In this view, we summarize the research progress in the new generation EGFR-TKIs after third-generation, in order to provide some reference for the follow-up research and treatment.

肺癌是世界范围内患病人数及癌症相关死亡人数最多的癌种^[1]^。2015年中国恶性肿瘤流行情况分析显示2015年我国新发肺癌病例及肺癌死亡人数分别约为78.7万例和63.1万例，均居于首位^[2]^。而美国2019年流行病学调查研究结果预测2019年美国新发肺癌228, 150例，占所有新发肿瘤的12.9%；新发肿瘤中肺癌死亡数为142, 670例，占所有新发肿瘤死亡率的23.5%^[3]^。

AURA3^[4, 5]^研究结果显示奥希替尼二线治疗获得性T790M突变的非小细胞肺癌（non-small cell lung cancer, NSCLC）患者，其与传统含铂双药治疗的无进展生存期（progression-free survival, PFS）分别为10.1个月*vs* 4.4个月，总生存期（overall survival, OS）分别为26.8个月*vs* 22.5个月。而FLURA Ⅲ期^[6, 7]^研究结果显示，奥希替尼作为一线治疗药物时，对比标准表皮生长因子受体酪氨酸激酶抑制剂（epidermal growth factor receptor-tyrosine kinase inhibitors, EGFR-TKIs）治疗局部晚期或转移性表皮生长因子受体（epidermal growth factor receptor, *EGFR*）突变型NSCLC，其PFS显著延长（18.9个月*vs* 10.2个月），OS则为38.6个月*vs* 31.8个月。虽然上述研究数据显示出了奥希替尼的优越性，但大多数患者在使用一段时间后仍出现耐药问题，故本文对三代后的新一代EGFR-TKIs研究现状进行综述。

## 1 第三代EGFR-TKIs耐药机制简述

虽然EGFR-TKIs靶向药的出现极大地提高了突变型NSCLC患者的生存期，但因其会产生耐药性的特质仍然为临床工作者带来挑战。故对第三代TKIs耐药机制进行简述：*EGFR*再突变耐药机制中，目前占比最高的耐药机制为C797S突变^[8]^，此突变与T790M在等位基因上的位置决定后续治疗的选择。且研究表明奥希替尼一线治疗晚期*EGFR*突变NSCLC时，C797S突变频率为7%；而当奥希替尼二线治疗晚期*EGFR*突变NSCLC时，C797S突变频率则为14%。另外，L718Q、L844V、G796D/S/R、L792F/H/Y、G724S等罕见突变及后续治疗目前也有研究指出，详见表 1^[7-20]^。另外，T790M减少或缺失、*EGFR*扩增^[15]^均可引起耐药现象。

**表 1:** *EGFR*再突变及相关治疗的临床研究 Clinical researches of *EGFR* re-mutation and related treatment

Study	Mutation site	T790M mutation	Treatment
Ramalingam *et al*^[7]^	C797S	On the same allele^[9, 10]^	EAI045 with cetuximab
Papadimitrakopoulou *et al*^[8]^	C797S	On the separate alleles^[9-11]^	First-generation in combination with third-generation EGFR-TKIs
Bersanelli *et al*^[12]^	L718Q	Deletion^[13-15]^	Osimertinib, gefifitinib, afatinib
		Existence^[13]^	The mutation is resistant to all drugs
Ercan *et al*^[13]^	L844V	Deletion^[13]^	Osimertinib, gefifitinib, afatinib
		Existence^[13]^	The mutation is resistant to all drugs
Ou *et al*^[16]^	G796D/S	-	-
	G796R		
Zheng *et al*^[17]^	G796D/S	On the same allele^[18]^	EAI045 alone or in combination with cetuximab (The curative effect is very little)
	G796R		
Chen *et al*^[19]^	L792F/Y	-	-
	L792H	On the same allele^[18]^	EAI045 alone or in combination with cetuximab (The curative effect is very little)
Fassunke *et al*^[20]^	G724S	-	Afatinib
-: There is no indication in the literature; EGFR-TKIs: epidermal growth factor receptor-tyrosine kinase inhibitors.			

EGFR非相关耐药机制中，研究指出：间质表皮转化（mesenchymal-epithelial transition, *MET*）和人表皮生长因子受体2（human epidermal growth factor receptor 2, *HER2*）的扩增^[8]^、*RAS*突变^[21]^、*BRAF*突变、1-磷酸酰肌醇-3-激酶（phosphatidylinositol 3-kinase, *PIK3CA*）突变、磷酸酶和张力蛋白类似物（phosphatase and tensin homolog, PTEN）缺失^[9]^、胰岛素样生长因子受体1（insulin-like growth factor 1 receptor, IGF1R）上调^[22]^、成纤维细胞生长因子受体（fibroblast growth factor receptor, FGFR）信号通路异常^[8]^、小细胞肺癌（small cell lung cancer, SCLC）转化^[23]^、上皮间质细胞转化^[24]^（epithelial mesenchymal transition, EMT）、细胞周期基因改变^[8, 9]^、致癌基因融合^[8, 9]^、Bcl-2样蛋白11缺失、极光激酶A的激活、Src通路激活、整合素-kras复合物的形成、AXL的激活、EPHA2过表达^[25-31]^等均导致耐药。

## 2 三代后的新一代EGFR-TKIs研究进展

### 2.1 EAI045

Jia等^[10]^在用纯化的EGFR L858R/T790M激酶筛选出的化合物文库中，鉴定出了一种新的表皮生长因子受体变构抑制剂-1（EAI001），其对*EGFR* L858R/T790M突变有活性。进一步的优化得出EAI045化合物。对EAI045细胞活性的研究表明，在L858R/T790M突变的NSCLC细胞系H1975、稳定转染L858R/T790M突变体的NIH-3T3细胞中，EAI045有效地降低了EGFR自磷酸化，但不能完全消除EGFR自磷酸化。在一组*EGFR*突变的Ba/F3细胞中显示，EAI045抑制L858R/T790M和L858R突变细胞的增殖，但不抑制外显子19del/T790M或亲本Ba/F3细胞的增殖。进一步在EAI045与EGFR二聚体干扰抗体-西妥昔单抗的联合使用中发现，联合治疗可抑制L858R/T790M EGFR Ba/F3细胞的增殖以及L858R/T790M小鼠肿瘤的显著消退，而在携带19del/T790M EGFR外显子的小鼠中没有治疗反应。且研究表明当西妥昔单抗浓度为10 μg/mL时，EAI045抑制L858R/T790M EGFR Ba/F3细胞的半数抑制浓度（50% inhibitory concentration, IC_50_）约为10 nmol/L。同样的，在L858R/T790M/C797S Ba/F3细胞、L858R/T790M/C797S突变的小鼠中，EAI045与西妥昔单抗联合使用也观察到类似结果。然而，这一联合治疗方式目前没有在人体中进行过试验，仍需要大量的研究工作。

### 2.2 JBJ-04-125-02

To等^[32]^使用迭代过程合成了EAI001的结构类似物-化合物JBJ-02-112-05，其在异吲哚酮部分上加有5-吲哚取代基，进一步的优化得到化合物JBJ-04-125-02，它将EAI045的2-羟基-5-氟苯基和异吲哚酮上的苯基哌嗪结合在一起。在一组稳定转染*EGFR* L858R、*EGFR* L858R/T790M或*EGFR* L858R/T790M/C797S突变的Ba/F3细胞株中测试表明，与EAI045联合/不联合西妥昔单抗相比，JBJ-04-125-02联合/不联合西妥昔单抗是最有效的，且其是唯一可以作为单一药物抑制细胞增殖的化合物，且JBJ-04-125-02抑制上述三种突变的IC_50_分别为1.0 nmol/L、0.4 nmol/L-0.5 nmol/L和0.0 nmol/L-0.1 nmol/L。但是JBJ-04-125-02则对亲本细胞Ba/F3、野生型EGFR Ba/F3细胞和*EGFR* 19外显子突变的Ba/F3细胞生长无抑制作用。在*EGFR* L858R/T790M/C797S基因工程小鼠体内的研究表明，JBJ-04-125-02与JBJ-02-112-05均可抑制肿瘤的生长，但JBJ-04-125-02的效果较好。后续的研究表明，JBJ-04-125-02与奥希替尼联合使用，增强了JBJ-04-125-02与突变型*EGFR*的结合，更加有效地抑制细胞生长，促进细胞凋亡。这一结果提示，对于*EGFR*突变型肺癌患者，联合使用共价突变选择性ATP竞争抑制剂和变构EGFR抑制剂可能是一种有效的治疗方法。

### 2.3 2, 9-二取代8-苯硫基-9H-嘌呤化合物

Hei等^[33]^研究合成了31个2, 9-二取代8-苯硫基/苯亚砜/苄硫基-9H-嘌呤衍生物，其中2, 9-二取代8-苯硫基-9H-嘌呤类化合物-C类分为C1-C12，结果显示化合物C1、C2和C3对HCC827细胞系中等活性，随着取代基体积的增加，化合物C4、C5和C6的抗增殖作用明显增强。而相比较A549细胞系，除了化合物C4和C6对A549细胞系具有中等的抑制增殖作用外，其余受试化合物对H1975和A549细胞的增殖抑制作用较弱。另外，R2位含有4-N, N-二甲氨基哌啶-1-基的化合物C9对EGFR L858R有较强的抑制作用，其对HCC827细胞的选择性是A549细胞的340倍，且在5.0 mg/kg剂量下对已建立的裸鼠HCC827移植瘤模型有明显的抑制作用。实验表明2, 9-二取代8-苯亚砜-9H-嘌呤类化合物D、2, 9-二取代8-苄基硫代/苄基磺酰基-9H-嘌呤类化合物E/F与化合物C对HCC827的抑制作用均较差。但含有8-（4-氟苯硫基）部分的化合物C12则表现出对EGFR L858R/T790M/C797S的中等抑制活性，IC_50_为114 nmol/L。这一结果提示化合物C12的后续优化可能成为三代后的新一代EGFR-TKIs。

### 2.4 2, 4, 6-三取代吡啶并[3, 4-d]嘧啶化合物

有研究^[34]^用6-苯氨基-吡啶[3, 4-d]嘧啶取代EGFR-TKIs的药物支架2-苯氨基嘧啶或2-苯氨基杂环-嘧啶骨架获得新的EGFR-TKI，利用支架跳跃原理设计出化合物A。进一步对化合物A1、A2的测试表明，这两个化合物对HCC827细胞没有明显的抑制增殖活性，猜测这一现象可能为吡啶并[3, 4-d]嘧啶的6位氢原子与苯胺的2位氢原子之间的空间位阻使其不可能维持药理构象导致。为了克服这一问题，研究设计了化合物B-吡啶环取代苯环，吡啶-2-氨基-吡啶[3, 4-d]嘧啶支架与铰链区残基Met793之间可以形成双齿氢键，从而相互作用。与化合物A1和A2相比，优化后的化合物B1、B2、B4对HCC827、H1975和A549细胞表现出明显的抗增殖活性，而化合物B7（引入甲基）、B12和B13（引入三氟甲基）对HCC827细胞的杀伤活性显著降低。化合物B29-B31因为其吡啶[3, 4-d]嘧啶骨架的4位上连接了一个羟基取代物，而羟基可能与EGFR的磷酸结合位点（phosphate buffered saline, PBS）形成的额外氢键相互作用，所以具有提高抗增殖的作用，且化合物B30对三重突变体*EGFR* L858R/T790M/C797S具有显著的抑制作用，IC_50_为7.2 nmol/L。一系列的实验表明，吡啶并[3, 4-d]嘧啶类支架的2位苯氨基、4位4-羟基基环己基和6位5-[4-（二甲基氨基）哌啶-1-基]-吡啶-2-基有利于化合物的抗增殖作用。故2, 4, 6-三取代吡啶并[3, 4-d]嘧啶衍生物中化合物B30可以进一步优化，以便成为新一代EGFR-TKIs。

### 2.5 2-芳基-4-氨基喹唑啉化合物

Park等^[35]^以2-芳基-4-氨基喹唑啉为分子核心设计发现了对del746-750/T790M/C797S突变体有效和特异的新一代EGFR抑制剂，其中化合物1对该突变体具有较强的酶抑制活性，且选择性是野生型EGFR的163倍。模拟研究表明，化合物1的末端酚基分别与Met793的主链酰胺氮和Gln791的氨基羰基氧接收和给予氢键，且这两个氢键受到化合物1的苯环和Met790侧链之间相邻的疏水作用支持。另外，化合物1中还存在Ser797的侧链羟基和1的吡啶-3-甲胺基团之间形成的两个氢键。正是由于这些氢键，才使得Ser797取代Cys797成为EGFR-TKIs的第三个耐药突变。化合物1的末端酚类部分1的对位指向一个包括Val726、Met 790、Th854、Lys 745和Asp855的结合口袋，而这个结合口袋则是*EGFR*突变体的易受攻击目标。在该位置上分别引入氰基、甲酯，合成化合物10、13、19，这三种化合物对del746-750/T790M/C797S突变体的选择性是野生型的1, 000多倍，IC_50_分别为23.7 nmol/L、46.7 nmol/L和17.9 nmol/L。总而言之，有效的化合物与突变残基Met790和Ser797形成的强烈的相互作用可以用来解释相关化合物对三重突变体的抑制活性。

### 2.6 4-氨基吡唑嘧啶类化合物

Engel等^[36]^在研究将Bruton酪氨酸激酶（Bruton's tyrosine kinase, BTK）抑制剂4-氨基吡唑并嘧啶支架作为一类新型*EGFR* T790M突变抑制剂时，合成了1a-1c三种化合物。这三种化合物均对携带*EGFR* L858R/T790M耐药的非小细胞肺癌细胞株H1975有较好的抑制效果，而对野生型EGFR细胞株A431的抑制效果较差。进一步的研究表明，化合物1c对获得性耐药L858R/T790M/C797S显示出中等抑制作用，IC_50_为88 nmol/L。其亲和力约是对*EGFR* L858R/T790M/C797S没有明显作用的1a和1b的20倍。这一结果提示4-氨基吡唑并嘧啶支架可能是后续药物研发的机制之一。

### 2.7 三取代咪唑类化合物

Günther等^[37]^在高效可逆p38抑制剂的基础上设计出了三取代咪唑类化合物。这类化合物对*EGFR* L858R/T790M/C797S三重突变体有抑制作用。实验表明，化合物11d/e/h对野生型*EGFR*、L858R突变型*EGFR*的IC_50_值均低于0.5 nmol/L，对L858R/T790M突变型*EGFR*的IC_50_值分别为12.5 nmol/L、8.4 nmol/L和6.6 nmol/L，而对L858R/T790M/C797S三重突变型*EGFR*的IC_50_值则分别为7.64 nmol/L、8.8 nmol/L和21.1 nmol/L。研究基于p38 mitogen-activated protein kinase（MAPK）抑制剂的靶外打击以及后续操作，合成了一系列具有7-氮杂环己烷铰链结合基序的三取代咪唑类化合物，且对化合物11的分子模拟表明，其可与ATP结合位点可逆性结合，在此基础上设计出了对L858R/T790M/C797S三重突变体等效的低纳米抑制剂11d和11e，这些化合物不需要形成共价键就可以获得高效力。这些研究有力地支持了三取代咪唑类化合物作为三代后的新一代EGFR抑制剂克服三重突变的可能性。

### 2.8 吡咯嘧啶类化合物

Lategahn等^[38]^将吡咯嘧啶类支架作为新型、突变型选择性共价抑制剂进行了一系列检测，合成了第一组吡咯嘧啶类EGFR抑制剂（包括12种化合物，并对其溶解性和细胞活性进行了优化）。且这类支架利用Mitsunobu反应进一步衍生得到了容易分离的3-取代吡咯嘧啶-4-酮和4-取代吡咯嘧啶的混合物，并合成了第二组吡咯嘧啶类EGFR抑制剂。研究表明共价键的形成可有效抑制突变体的活性。依据这一现象，研究者设计了化合物17a（第一组吡咯嘧啶类EGFR抑制剂的一种），其对EGFR L858R、EGFR L858R/T790M的IC_50_分别为2.3 nmol/L和4.0 nmol/L，且对野生型EGFR有选择性（IC_50_为15 nmol/L）。Western blot显示17a可抑制EGFR及其下游级联蛋白的磷酸化水平。第二组吡咯嘧啶类EGFR抑制剂包括N型烷基化吡咯嘧啶类化合物29a-l、O型烷基化吡咯嘧啶类化合物19a-h，这两类化合物均与EGFR中的Cys797共价结合，具有可逆结合特性。前者对*EGFR* L858R/T790M/C797S突变的IC_50_值> 110 nmol/L，而后者的IC_50_值< 50 nmol/L。其中活性最强的是化合物19 g、化合物19 h（在4位上分别含有异丙基醚和异丁基醚），IC_50_值均约为9 nmol/L，且两者可以与双突变体*EGFR* T790M/C797S形成配合物。研究指出N型和O型烷基化吡咯嘧啶类化合物有较高的抗EGFR活性，与第一、二、三代EGFR-TKIs相比，上述化合物具有更好的生化效力。因此吡咯嘧啶类化合物可作为新一代EGFR抑制剂的研究方向。

## 3 展望

第三代EGFR-TKIs虽然有一定的治疗效果，但因其不可避免出现的耐药性，且存在异质性，导致仍有一部分耐药机制未知。以耐药机制为切入点，并将其作为相应靶点从而研制出新的治疗药物。虽然目前临床前研究已出现几种有效的可被称之为三代后的新一代EGFR-TKIs，但是尚未在人体中进行相关试验。此外，三代EGFR-TKIs耐药机制不仅包括*EGFR*的再突变，还包括更复杂的耐药机制如：MET、RAS等通路的活化、EMT及病理类型的转化等。因此，除抑制EGFR信号通路内的耐药突变外，联合治疗可能也是克服EGFR通路外耐药的选择方法之一。
